# Fully-Enclosed Ceramic Micro-burners Using Fugitive Phase and Powder-based Processing

**DOI:** 10.1038/srep31336

**Published:** 2016-08-22

**Authors:** Truong Do, Changseop Shin, Patrick Kwon, Junghoon Yeom

**Affiliations:** 1 Department of Mechanical Engineering, Michigan State University, East Lansing, MI, USA

## Abstract

Ceramic-based microchemical systems (μCSs) are more suitable for operation under harsh environments such as high temperature and corrosive reactants compared to the more conventional μCS materials such as silicon and polymers. With the recent renewed interests in chemical manufacturing and process intensification, simple, inexpensive, and reliable ceramic manufacturing technologies are needed. The main objective of this paper is to introduce a new powder-based fabrication framework, which is a one-pot, cost-effective, and versatile process for ceramic μCS components. The proposed approach employs the compaction of metal-oxide sub-micron powders with a graphite fugitive phase that is burned out to create internal cavities and microchannels before full sintering. Pure alumina powder has been used without any binder phase, enabling more precise dimensional control and less structure shrinkage upon sintering. The key process steps such as powder compaction, graphite burnout during partial sintering, machining in a conventional machine tool, and final densification have been studied to characterize the process. This near-full density ceramic structure with the combustion chamber and various internal channels was fabricated to be used as a micro-burner for gas sensing applications.

Miniaturization of chemical system has garnered significant attentions in chemistry and biology due to many advantages such as enhancement in heat/mass transfer rates at small scale, reduction in expensive reagents and hazardous wastes, and facilitation of massive parallelization in reaction/catalyst screening and optimization^1^,^2^,^3^. The significant technological advancements for micro-chemical systems (μCSs) have been focused on chemical reactions, separation, and sensing in a low-to-medium temperature range (20 °C to 600 °C). One notable example is a lab-on-a-chip or micro total analytical system, in which the total sequence of laboratory processes is integrated to perform chemical synthesis, transport, and analysis, and it has profound influence in chemistry and biomedical areas^4^,^5^. In some cases, the microreactors and heat exchangers in μCS need to be operated at high temperatures (>600 °C) and/or under highly corrosive environments like solid-oxide fuel cells^6^,^7^,^8^,^9^, fuel reformers^10^,^11^,^12^, combustion burners^13^,^14^, and gasifiers^15^,^16^. However, high-temperature μCSs with sophisticated design and similar level of integration found in low-temperature counterpart have rarely been realized mainly because the conventional μCSs such as silicon, glass, polymers, metals and conventional metal alloys are not stable at these high operating temperatures. Ceramic materials offer excellent high-temperature compatibility and corrosion resistances, but pose significant manufacturing challenges due to their hardness and brittleness.

Several groups have demonstrated the promise of ceramic-based microreactors for medium-to-high temperature reactions such as hydrogen production from continuous reforming of propane^10^,^17^,^18^, oxidative coupling of methane^19^,^20^, catalytic combustion^21^, and nanoparticle synthesis^22^. One of the paramount challenges in fabricating ceramic μCSs is that the microfabrication techniques borrowed from well-established microelectronics and microelectromechanical system (MEMS), which are very effective for silicon- or polymer-based μCSs, are not compatible with ceramic materials. Instead, the conventional and non-conventional ceramic processing techniques have been utilized to create ceramic microreactors and other components of μCSs. These techniques include rapid prototyping using low-pressure injection molding^19^,^23^, micromachining^20^,^24^, sol-gel/nanoparticle casting^25^,^26^, and tape casting^27^,^28^. In the rapid prototyping process, a negative silicone mold is first created from the original polymer mold fabricated by micro-steoreolithography, which is used directly for low-pressure injection molding^29^,^30^. The resolution and surface quality of the ceramic components depend on the stereolithography quality of the original polymer mold^23^, and the critical dimension of hundreds of microns (which is a relevant length scale in most μCSs) can be easily obtained. More recently, the smallest feature size on the order of a few microns in ceramic structures has been fabricated using the soft-lithographic molding technique like micromolding in capillaries combined with sol-gel casting^26^,^31^. However, these molding/casting-based techniques can create only the open channel or chamber structures due to the demolding requirement. To utilize them in μCS applications, the fabricated structures need to be bonded with or packaged in another high-temperature material to form sealed microchannels or microreactors. Tape casting with low-temperature co-fired ceramic (LTCC) is perhaps the most widely used technique when it comes to the fabrication of the ceramic microreactors and microchannels^28^,^32^,^33^,^34^,^35^,^36^. While low co-firing/sintering temperature (<900 °C) is beneficial for integrating metal electrodes and other applications, the operation temperature is typically limited due to the presence of the glass phase. Unlike the various molding techniques, tape casting is capable of producing suspended structures, enclosed cavity or microchannels for μCSs. However, the suspended structures tend to deform and sag due to high lamination pressures and the softening of the glass component in the ceramic composite during sintering^35^. Multilayer lamination with fugitive materials such as waxes, polymeric materials, and carbon materials was used to support the embedded structures during lamination and sintering^37^. Wax- and polymer-based fugitive materials, however, were completely burnt out even before the sintering of LTCC, and therefore sagging of the suspended region cannot be prevented during sintering^38^. Moreover, in tape casting, each machined layer needs to be aligned to the previous layers before lamination, entailing some special equipment for alignment and lamination.

Here we propose a simple, inexpensive, reliable, and reproducible ceramic manufacturing technology for high-temperature μCSs and microdevice application. The proposed technique employs the cold compaction of metal-oxide powders with a graphite fugitive phase for the embedded features. Without a binder phase in the powder mix, the final chemistry and dimension of the sintered ceramic structures can be more precisely tuned. The advantages of the proposed powder-based technique include (1) a one-pot, cost-effective process to create either open or fully-enclosed ceramic microreactors and microchannels, (2) near-full density ceramic structures without any other phases (e.g. organic or glass materials) in the final devices, (3) partially-sintered ceramic structures facilitating machining, and (4) abilities to control the surface finish of the internal cavity walls and easily incorporate additional features on the cavity surface. The key process steps such as powder compaction, graphite burn-out during partial sintering, machining of partially sintered ceramics, and final densification have been investigated to optimize the process. As a proof-of-the-concept demonstration, a fully-enclosed ceramic structure with sub-millimeter internal cavities is used for micro-burner and micro flame ionization detector (μFID) applications.

## Results and Discussions

To demonstrate the feasibility of the proposed powder processing, we consider a micro-burner whose critical dimension is below 1 mm. The micro-burner design employed in this study was previously reported^39^ and adopted without major modifications. Unlike most ceramic processing techniques that start with ceramic powders or sols mixed with polymeric binder phases, our approach exploits cold compaction of raw powders while utilizing a graphite fugitive phase to create internal cavities and channels. The proposed fabrication scheme allows the fabrication of open and fully-enclosed cavities/microchannels, and both configurations will be considered here (see Materials and Methods for the detailed process). A prototype micro-burner device with an open ceramic structure covered with a transparent quartz top is employed to visualize and optimize the flame in the micro-burner before the fabrication of the micro-burner with the fully-enclosed combustion chamber. We base our initial development on a flat circular disc with a small thickness-to-diameter ratio (~ 0.1) because the thin circular disc is one of the simplest shapes known to successfully compact the powder in a uniform density under a uniaxial load.

### Fabrication Results

**Figure 1:**
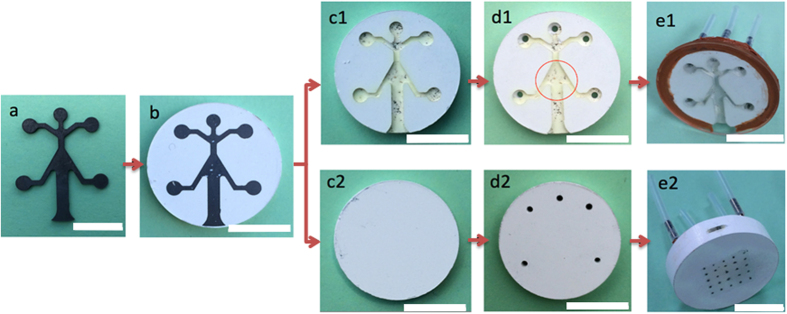
Photographs of (**a**) a 0.9-mm-thick CNC-machined graphite layer; (**b**) the graphite layer inserted into an Al_2_O_3_ powder bed; (**c**) the rest of the powder being applied (only for c2), compacted, and the partially sintered, removing the graphite fugitive phase; (**d**) holes drilled on the partially-sintered alumina devices; (**e**) fluidic tubing connected for micro-burner testing. Scale bar in all images is 1 cm.

We have successfully fabricated the alumina micro-burners in both open-channel and embedded-channel configurations. Figure 1 shows the photographs of the micro-burner samples in each process stage. It is shown that a 0.9-mm-thick graphite layer was CNC-machined (Fig. 1a) and sandwiched/cold-pressed into the alumina powder without damage (Fig. 1b). The fabrication result for the open-channel micro-burner with the quartz top is shown in the top row in Fig. 1 (Fig. 1c1–e1) while the fully-embedded micro-burner in the bottom row (Fig. 1c2–e2). For the fully-embedded sample, the other half of the alumina powder was deposited and compacted to what is shown on Fig. 1b. Figure 1c1 indicates that the graphite layer was completely burn out during the partial sintering at 800 °C as the decomposition temperature of the graphite phase is below 800 °C. In case of the fully-enclosed sample, whether or not the graphite layer was completely removed was not clear by the visual inspection. We will address this issue in more details with the sintering characterization results in the following section. The partially-sintered state of the alumina allows us to use the conventional machining (e.g. milling, drilling, etc.) for further modification. The holes were drilled in the partially-sintered alumina burner for fluidic connections (see Fig. 1d). The medium-temperature epoxy (red color on Fig. 1e1) was used to seal the open-channel micro-burner (between the 25-mm dia. quartz disc and the 22-mm dia. alumina disc). Stainless Steel (SS) tubing fixed with the high-temperature epoxy provides gas-tight delivery of fuel (e.g. hydrogen) and oxidant (e.g. oxygen and/or air) to the burner cavity.

**Figure 2:**
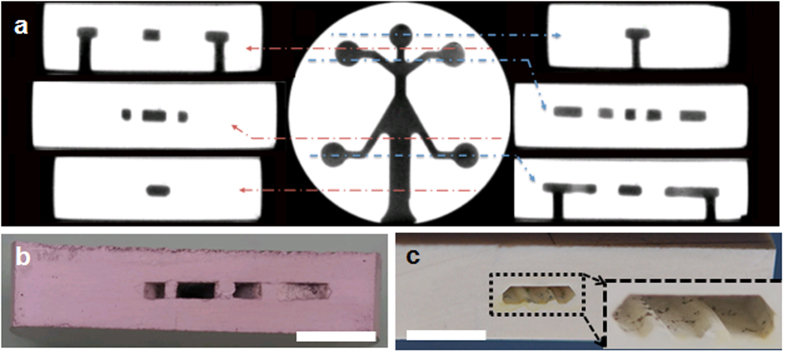
Images of the internal cavity and channels: (**a**) X-ray micro-computed tomography (micro-CT) for the fabricated alumina micro-burner with various cross-sectional views; photographs of the cross-sectional views for (**b**) the same micro-burner and (**c**) the submillimeter internal channel with the additional features on the wall. Scale bar for (**b**) and (**c**) is 5 mm.

Figure 2 shows the cross-section views of the fully-sintered micro-burner with the embedded channel configuration. We used x**-**ray micro-computed tomography (micro-CT) technology to noninvasively image the internal cavity and channels (see Fig. 2a). This technique can be particularly useful to orient and position the internal cavity and channels of the fully-enclosed structure for drilling or other machining process of external features. It is shown that the internal features took the exact shape of the graphite phase and the machined holes were well aligned to the internal channels. The photograph of the crosscut sample reveals that no noticeable cracks or defects were found (see Fig. 2b). Performing the micro-CT scan each time to locate the internal feature can be costly. Alternatively, we modified the design by extending the exhaust part of the graphite and exposing it to the exterior surface, which allowed us to find the internal channels for machining. Another unique aspect of this technique is an ability to incorporate additional structures on the channel wall without additional process steps. For example, Fig. 2c shows two protruded lines of the trapezoidal cross-section at the bottom of the submillimeter channel embedded in the alumina device. This was done by simply making two grooves in the graphite fugitive phase before compaction. This type of the structure would be difficult to be fabricated within the cavity walls using any other ceramic processing technique discussed in Introduction. Moreover, the surface finish quality of internal cavities can be tuned as it is directly related to the surface finish of the fugitive phase (see Supplementary Information Figure S1).

### Processing Characterization

We utilized the *fully-enclosed* design of the alumina micro-burner for the process characterization including powder compaction, optimization of the sintering temperature profile, and thermogravimetric analysis (TGA) experiments for graphite burn-out. One of the most critical steps in the proposed fabrication scheme is compaction. The presence of the stiff graphite of a complex shape impedes the powder flow, frequently causing non-uniform density distribution and resulting in cracks in the final component. One simple remedy is to remove sharp edges and geometric complexities in the machined graphite layer. The rounded edges and corners facilitate the powder to flow around them, preventing undesirable cracks due to the non-uniform density distribution. The edges of the graphite after cut in a CNC machine were smoothened by manual grinding. In addition, the sharp protrusion or the feature of graphite with a high aspect ratio makes the resulting powder structure susceptible to crack formation during full sintering because of the non-uniform density distribution. For example, the region (circled in Fig. 1d1) intersecting two gas channels to the combustion chamber was found to be prone to the crack formation. Moreover, if the size of the graphite fugitive phase is too large in comparison to the overall ceramic sample, the ceramic sample may become collapsed or fractured. Since the powder holds its shape after compaction with the friction among the powder, the sufficient powder area is needed in terms of both thickness and planform area.

**Figure 3:**
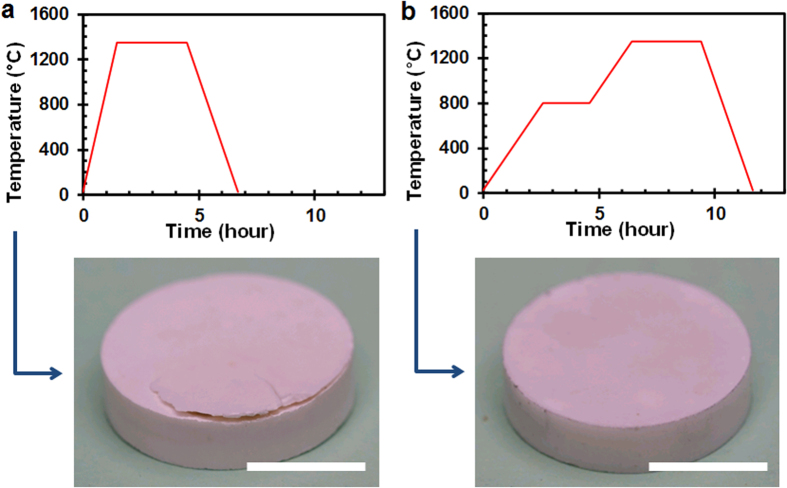
Sintering temperature profile affecting the micro-burner structure with the internal cavity: (**a**) a single ramp-up in temperature bursting out the structure (see text); (**b**) adding a 2-hour-long soaking step at 800 °C, allowing the graphite fugitive phase to get oxidized before powder consolidation. Scale bar is 1 cm in both images.

In a typical LTCC process, the burning of fugitive materials requires large openings because ceramic tapes consisting of the ceramic matrix infiltrated with polymeric or glassy phases are non-porous, leaving little room for gas diffusion. Because we compact pure alumina powder without any binder phase, the powder compact is still substantially porous, allowing gases (e.g. O_2_ and CO/CO_2_) to diffuse in and out. Among many materials serving as fugitive phases^37^, we chose graphite for two reasons. First, graphite has a very low coefficient of the thermal expansion (2~6 μm m^−1^ K^−1^), which minimizes the stress exerted onto the powder compact during partial sintering. This is important because the ceramic powders are in an extremely fragile state when the graphite is burnt out. Also the dimension of the integrated cavity can be predicted with better accuracy compared to other polymer-based fugitive phases. Secondly, the graphite burns out before the alumina powder starts to consolidate. Adequate interstitial spaces are necessary for the byproducts of graphite oxidation, mainly CO and CO_2_, to escape. However, each graphite grade is slightly different in its oxidation temperature and a care must be taken in determining an appropriate temperature ramp rate and a soaking temperature and duration if necessary. Figure 3 shows the two different sintering temperature profiles (oven temperature vs. time) and the resulting alumina micro-burners. When a full sintering temperature was reached at a constant ramp rate (15 °C·min^-1^) without any soaking step, a crack was observed at the side or corner of the final structure (see Fig. 3a). A similar crack was observed even at lower ramp rates. We performed thorough visual inspection of the compacted green ceramics using a microscope and removed the ones with observable cracks due to the improper die pressing (see Supplementary Information Figure S2 for more images at each process stage).

Of course, there is still a possibility that cracks formed inside are not observable with visual inspection. In fact, the initial crack formation probably occurs at the edge or corner of the graphite during compaction. These preformed internal cracks can propagate to the exterior surface during the sintering process. We hypothesized that the crack propagation and structural integrity of the sintered ceramic structure would be significantly affected by the sintering temperature profile for the following reasons. On one hand, a higher ramp rate would induce a higher temperature gradient within the structure and in turn cause internal stresses to be developed, leading to crack propagation. On the other hand, an insufficient amount of time for graphite to completely burn out would generate a pressure build-up in the internal cavity as the consolidation of alumina powders progresses. We believe the former factor is less important since no crack was observed in the open-channel configuration regardless of the ramp rate. In the open-channel sample, the graphite phase is fully exposed to outer environment, and therefore there is no restriction for CO/CO_2_ to be released. Conversely, a competition between graphite volatilization and powder consolidation exists in the fully-enclosed sample. If the alumina powders become consolidated before all graphite phases are burnt out, CO/CO_2_ has little interstitial space to escape and the pressure will build up until the structure bursts open. This net increase in pressure within the cavity can be attributed to the different gas permeabilities of O_2_ and CO_2_ in porous metal oxide structures (see Supplementary Information). To facilitate graphite volatilization, we added a soaking step at 800 °C – the temperature high enough for graphite to burn out while low enough for alumina powders *not* to sinter (or consolidate) significantly. The modified sintering cycle allowed us to obtain the crack-free ceramic structures with the internal cavities (see Fig. 3b).

**Figure 4:**
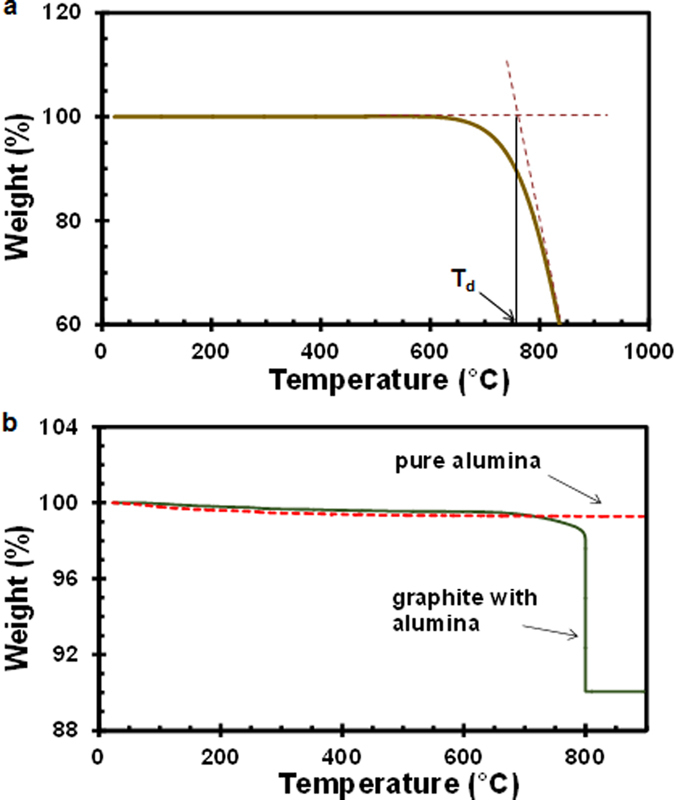
Thermogravimetric analysis (TGA) results: (**a**) a pure graphite sample; (**b**) a graphite piece embedded by and compacted with the alumina powder.

To understand the kinetics of the graphite burn-out with and without the presence of alumina powders, we conducted thermogravimetric analysis (TGA) on a small piece of graphite and a graphite piece embedded in the alumina powder compact. First, a pure graphite sample used in this study was tested to determine the onset temperature of decomposition (or oxidation). Figure 4a shows a percent weight change as a function of temperature for the pure graphite sample (~10 mg). The temperature was increased from room temperature to 850 °C at a constant ramp rate of 10 °C·min^−1^ with air flowing through the sample chamber. The weight loss started to take place at around 650 °C. The onset temperature (T_d_) of intense thermal decomposition can be determined by the intersection point of tangents to two branches of the TGA curve and be estimated to be approximately 760 °C (see Fig. 4a). This observation served as a basis for determining the soaking temperature of 800 °C in Fig. 4.

Next, the TGA experiment for the graphite piece embedded in the alumina powder compact was performed to model the graphite decomposition process in the fabrication of the micro-burner. A small (~7 mm in dia.) alumina compact encapsulating a graphite piece was fabricated in the same way that the micro-burner was made. The size of the graphite piece was scaled proportional to the alumina structure such that the mass and volume ratio of graphite to alumina remained the same as the original burner structure. The temperature profile used in the experiment was similar to the partial sintering step of the alumina micro-burner and consisted of (i) temperature rise from room temperature to 800 °C at the ramp rate of 15 °C·min^−1^, (ii) soaking at 800 °C for 2 hours, and (iii) ramping again to 900 °C at the ramp rate of 15 °C·min^−1^. The weight loss as a function of temperature is shown for the “graphite with alumina” sample in Fig. 4b. As expected, a noticeable decrease in weight was observed around 760 °C, corresponding to the onset temperature of the graphite decomposition. In the final ramping step (from 800 °C to 900 °C), no apparent weight loss was witnessed, suggesting that the entire graphite phase was completely burnt out during the 2-hour soaking step at 800 °C. After cooling down, the graphite/alumina sample was retrieved and inspected under the optical microscope. No crack was visible in the sample. Therefore, we conclude that the modified sintering schedule was capable of completely removing the graphite phase without damaging the ceramic structure. On a separate note, we unexpectedly observed a minute yet reckonable (~0.5%) decline in weight in the initial ramping step (from room temperature to 650 °C). Since no weight loss was seen in the pure graphite sample up to 650 °C, the alumina powder should be responsible for this initial weight loss. To understand this trend, we ran another testing only with the alumina powder of the same mass with the same operating condition and noticed the similar initial decrease in weight (see the red dotted curve in Fig. 4b). We exclude the possibility of the impurity inclusion as the alumina powder purchased has purity higher than 99.99% and was used in the as-received condition. Instead, we attribute this behavior to the hygroscopic nature of alumina powder – consisting of high-surface-area sub-micron-particles that absorbed moisture from ambient. Upon heating, these bounded water molecules were desorbed, leading to the initial weight loss^41^.

**Figure 5:**
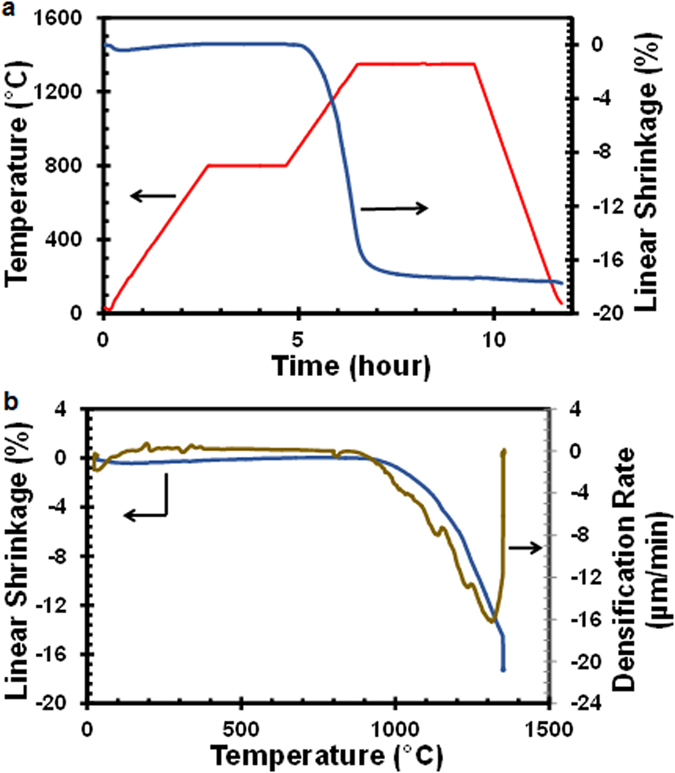
Thermomechanical analysis (TMA) results for compacted, pure alumina sample (3.8 mm in thickness): (**a**) linear shrinkage (blue) and temperature profile (red) plotted with time; (**b**) linear shrinkage (blue) and densification rate (green) plotted with temperature.

The linear shrinkage of alumina in air was measured by thermomechanical analysis (TMA) and is shown as a function of time and temperature in Fig. 5a,b respectively. The sintering temperature profile used in the TMA experiment was the same as the micro-burner sintering process. After an initial deep (which is related to the translation deformation induced by the load), there is a constant increase in displacement up to 800 °C, representing the thermal expansion of alumina powders. Because sintering had not occurred significantly below 800 °C, thermal expansion dominates over particle consolidation. In the 2-hour soaking period at 800 °C, the dimension of the sample did not change – the isothermal condition causing neither thermal expansion nor particle consolidation. Lack of shrinkage in the alumina sample at that temperature also indicates that the pores in the sample were still interconnected, allowing CO/CO_2_ emitted from graphite oxidation to escape without much barrier. The densification rate, a time derivative of displacement, is also plotted with temperature in Fig. 5b, showing that the temperatures for the onset of densification and the maximum densification rate are around 965 °C and 1,306 °C, respectively. It also suggests that sintering and densification essentially stopped at the temperature above 1350 °C. Finally, during the cool-down process, the sample was shrunk due to thermal contraction.

The TMA results were compared to the dimensional change of the micro-burner measured from the cross-sectional image (see Fig. 2). Table S1 shows the overall size and internal channel dimension of the alumina micro-burner at each process stage (see Supplementary Information). The green state (after compaction) of the sample had the diameter of 22.2 mm and the thickness of 4.98 mm. Just as the TMA shrinkage indicated, the sample size barely changed after the partial sintering at 800 °C. However, dimensional changes over 18% were made at the end of the full sintering process around 1,350 °C. The similar dimensional reduction was observed for the internal channel. We can also infer the dimensional shrinkage from the density (or volume) ratio of the green and sintered sample. In our process, the relative densities of the green and sintered ceramics were estimated about 52% and 96%, respectively. The volume ratio is then: V_sintered_/V_green_ = (1-*S*)(1-*S*)(1-*S*) = 52/96 = 54%, where *S* is a dimensionless shrinkage (assumed to be the same in all three directions). This leads to *S* ≈ 18.6%, which is consistent with the TMA results (maximum shrinkage rate of 18.3%) and the other reported values^42^,^43^,^44^. The volume ratio of 54% also suggests that the green-state ceramics (after compaction) are 40~50% porous. Since the TMA result indicates no significant shrinkage up to 1000 °C, the partially sintered alumina possesses the same level of high porosity, providing sufficient gas permeability for the graphite removal through the fully-enclosed structure and corroborating our earlier argument.

### Machining of Partially Sintered Ceramic (PSC)

It has been widely known that fully sintered ceramics including alumina in this study have poor machinability due to its stiffness and brittleness^45^. Machining green or white ceramic compacts^46^,^47^,^48^ would be easier if the final tolerance is not strict. Green machining is referred to as the machining of a ceramic in the unfired state, i.e., a powder compact before exposing to high temperature. In green machining, the powder is usually mixed with a binder phase (typically organic polymer or wax) to achieve the sufficient strength for machining. In this study, pure alumina powder was utilized without any binder phase. Therefore, the compacted powder was too difficult to handle and prone to fragment during machining, preventing us from performing green machining. On the other hand, white machining is an approach to machining on partially sintered ceramics (PSC)^48^. The powder compacts can be partially sintered by firing at a temperature substantially below their typical sintering temperature. The formation of necks among the individual powder particles during partial sintering provides PSCs with the strength to withstand during machining. Here we chose white machining to create fluidic connections to the enclosed channels in the micro-burner.

The extent of neck formation in PSCs, which determines the strength of the powder compact, highly depends on partial sintering temperature^49^. For this reason, the partial sintering condition such as pre-sintering temperature and its duration has been shown to significantly affect the quality of the machined features^45^. To understand the effect of partial sintering temperature on the machined features on PSCs, we prepared four alumina samples that were partially sintered at four different temperatures (600 °C, 800 °C, 1000 °C, and 1200 °C). The conventional machining processes such as drilling and milling were performed on these PSC samples using a small bench-top CNC machine. The quality of the machined features was correlated to the amount of chips and cracks generated around holes during machining. The optimal machining parameters (feed rate of 1 mm minneeds to be superscripted and cutting speed of 1500 rpm) for PSCs were determined based on the past work^45^ and used throughout the study.

**Figure 6:**
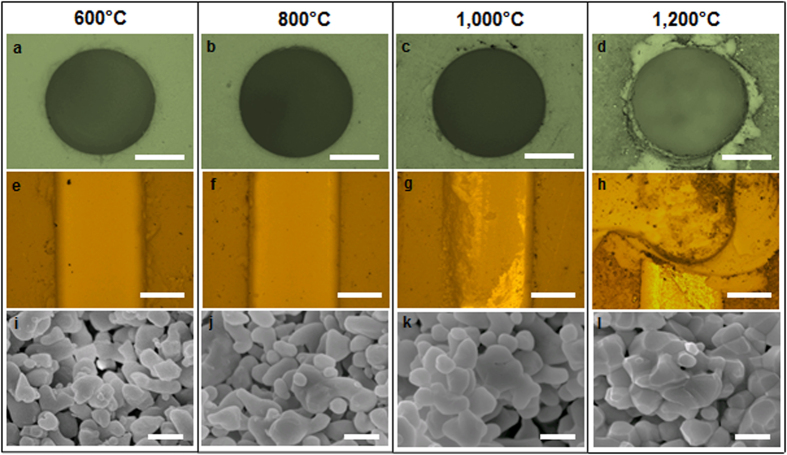
Effect of the partial sintering temperature (four different temperatures - 600, 800, 1000, 1200 °C) on the machined features of alumina PSCs: optical microscope images of (**a**–**d**) 1 mm diameter drilled holes and (**e**–**h**) 1 mm wide milled grooves; (**i–l**) SEM images of the corresponding microstructures. Scale bars are 0.5 mm for (**a**–**h**) and 200 nm for (**i**–**l**).

Figure 6 shows the effect of the partial sintering temperature on the machined features of alumina PSCs. The alumina sample partially sintered at 800 °C exhibited no pronounced surface chipping or cracks around the edges of the holes and grooves (see Fig. 6b,f). Minor cracks and chips were observed in the sample partially sintered at 600 °C (Fig. 6a,e) while more noticeable defects were seen for the 1000 °C sample (Fig. 6c,g). The sample partially sintered at 1200 °C exhibited the extensive chipping damages around the edges of the machined features (Fig. 6d,h). X-ray diffraction (XRD) analysis on the green (unfired) ceramic and these four PSCs suggests that the starting alumina powder is in the α-phase and there is no phase change during partial or full sintering (see Supplementary Information Figure S3). Therefore, the marked differences in the machined features of the alumina PSCs partially sintered at the different temperatures are not likely coming from the phase change of alumina. Unlike metals whose major removal mechanism is a local shear deformation, the underlying mechanism of machining PSCs is related to the breakage of necks between particles. When the partial sintering temperature increased from 600 to 800 °C (i.e., initial stage of partial sintering), more neck formation occurred, providing stronger connections among individual particles and more resistance to chipping or cracks. However, once the partial sintering temperature increased to 1000 °C or above, the consolidation taking place beyond the neck formation were too extensive such that brittle fracture became a dominant material removal mechanism. The high-resolution scanning electron microscopy (SEM) images in Fig. 6(i–l) show the extent of the neck formation and particle consolidation (or lack of porosity) for these four samples, corroborating our mechanistic explanations. Extensive neck formation on the powder compact can be clearly observed for the samples with higher partial sintering temperatures. To further characterize the mechanical properties of the PSCs sintered at different temperatures and correlate them to the observed machining behaviors, we conducted a 3-point bending test to measure the flexural strength of the PSCs. Figure S4 clearly shows the higher flexural strength with the increasing partial sintering temperature (see Supplementary Information). Combined with the results shown in Fig. 6, we can conclude that the PSC sample partially sintered at 600 °C was too fragile to machine while the sample partially sintered at 1200 ^°^C was strong but too brittle to machine.

### Micro-burner Testing

**Figure 7:**
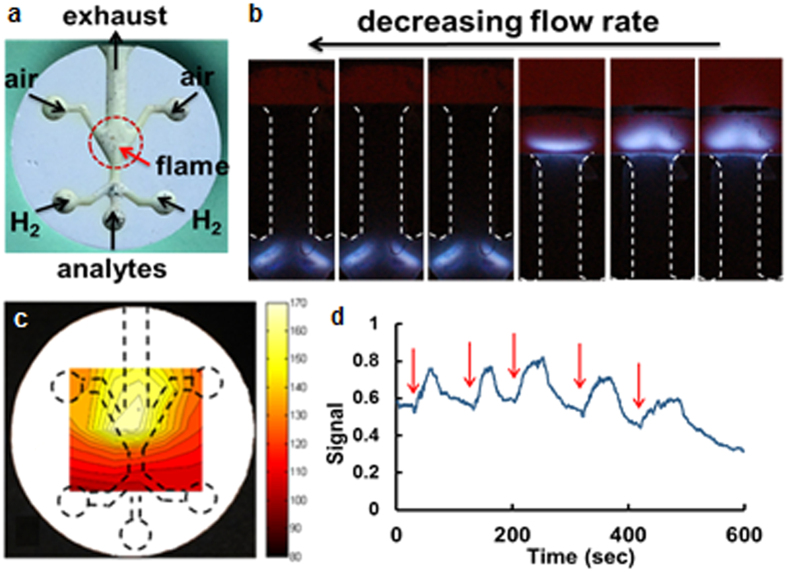
Micro-burner testing: (**a**) a photograph of the open-channel alumina micro-burner (18 mm in diameter); (**b**) flame characterization - reduction in a H_2_ flow rate anchoring a flame in the micro-burner; (**c**) a temperature distribution of the embedded micro-burner, indirectly representing the flame location; (**d**) signals collected upon natural gas injection after the micro-burner configured as a micro flame ionization detector (red arrows indicating the injection events).

The fully-sintered alumina sample fabricated in this study was tested for micro-burner applications. Both open-channel and embedded-channel configurations were experimented for visualization and proof-of-the-concept purposes, respectively. Fig. 7a shows how the fuel (H_2_) and oxidant (O_2_ or air) streams are connected to the micro-burner as well as the location of the exhaust. In essence, hydrogen generated by the electrolyzer^40^ and air from a miniature gas pump were fed to the open-channel micro-burner (bonded with a quartz window, see Fig. 1e). Under the laminar flow condition, these two streams create a stable hydrodynamic boundary layer in the flame region where a folded diffusion flame would be formed^39^. When the H_2_ flow rate was high (~100 sccm), the flame was ignited at the outside of the exhaust channel (see Fig. 7b). As the H_2_ flow rate (controlled by the electrical power applied to the electrolyzer) was reduced to 30~50 sccm, the flame started to move into the exhaust channel and eventually became anchored in the cavity. In order to reduce the amount of air that needed to be pumped, we added an O_2_ flow co-generated by the electrolyzer to the oxidant stream. The oxidant flow rate (O_2_ plus air) was determined to keep the fuel-to-oxidant ratio stoichiometrically correct or under lean-burn condition. A stable folded flame was observed for the range of the hydrogen flow rates (40~55 sccm) and oxidant flow rates (20~27 sccm for oxygen and 20~80 sccm for air).

After we identified the H_2_ and air flow rates that anchored the flame inside the combustion chamber, we tested the enclosed micro-burner with the similar flow conditions. The flame generated within the enclosed micro-burner cannot be observed due to the opaque nature of the alumina walls. Therefore, we indirectly verified the presence and approximate location of the flame by recording the outer wall temperature of the micro-burner. Fig. 7c depicts the temperature distribution of the burner’s exterior surface measured by the thermocouple from 7 by 7 points (49 total measurements with each point 1.5 mm apart, see Supplementary Information Figure S5a). The region of the highest temperature indicates the location of the flame, which resembles the flame location of the quartz/alumina micro-burner. The maximum steady-state temperature of the alumina outer surface was measured to be around 170 °C (for flow rate conditions: H_2_ 35 sccm and O_2_ 17.5 sccm/air 20 scccm). This temperature is much lower than the adiabatic flame temperature of the oxy-hydrogen flame, which is between 2210 and 3200 °C depending on the fuel-to-oxidant ratio and oxygen-to-air ratio in the oxidant stream. The dependence of the temperature distribution on different flow rate conditions are also shown in Supplementary Figure S5. The low thermal conductivity of alumina (12~38 W·m^−1^ K^−1^) therefore provided reasonable thermal isolation compared to the other common micro-burner materials like metals or silicon. The enclosed micro-burner was continuously operated for more than 6 hours with the stable flame throughout. More than 10 micro-burners have been tested over the 10-month period, and no structural damage has been observed due to the high operation temperature or repeated heating/cooling (i.e. thermal stresses) from each run.

Finally, the alumina micro-burner was tested as a micro flame ionization detector (μFID). Two tungsten wires (0.5 mm diameter) were inserted into the exhaust to serve as electrodes. A flame was ignited and anchored into the micro-burner cavity with the flow rate conditions of H_2_ 45 sccm and O_2_ 22.5 sccm/air 20 scccm. This oxy-hydrogen flame ionizes hydrocarbon molecules, and the produced ions are driven by the applied electric field and collected by the electrodes. Without further optimization, we injected a train of 0.1 mL of natural gas through the analyte port using a gas-tight syringe (red arrows in Fig. 7d). With the applied voltage of 160 V between the electrodes, a generated current was converted to an amplified voltage via a transimpedance amplifier (gain = 10^5^). Each peak in Fig. 7d corresponds to each event of natural gas injection. Due to large dead volumes associated with the injection port and the manual syringe injection setup, the obtained signals were short and broad. The electrode design and operation conditions were far from being optimal, and therefore the further characterization of the μFID would significantly improve the device performance. Though the performance of the μFID does not quite match that of the state-of-the-art system, this testing demonstrates a practical use of the micro-burner created by the proposed ceramic processing.

## Conclusions

In this paper, we presented a unique ceramic powder processing method to fabricate the ceramic structures with internal cavities and channels for microchemical system applications. High-purity, binder-free alumina micron-powder was compacted with the graphite fugitive phase embedded in the powder bed. The graphite was later burnt out during partial sintering, forming the cavity and channels. The sintering schedule used in partial (and full) sintering critically influenced the structural integrity of the final alumina structure. Instead of the continuous temperature ramping to the full sintering temperature, the compacted alumina was partially sintered at 800 °C for two hours, which not only facilitated the removal of the graphite fugitive phase but also promoted the optimal neck formation for the subsequent machining processes. The TGA and TMA results showing graphite oxidation and alumina densification kinetics supported the competing nature of graphite burn-out and powder consolidation. The quality of the machined features on the partially sintered alumina was investigated using various imaging techniques, revealing that the partial sintering temperature is an important parameter for machining. Finally, the fabricated open-channel and fully-enclosed alumina micro-burners were tested in various flow rate conditions of hydrogen and oxygen/air, demonstrating that the fully-enclosed device functioned as designed without failing over long-term and cyclic operations.

## Materials and Methods

### Fabrication Procedure

**Figure 8:**
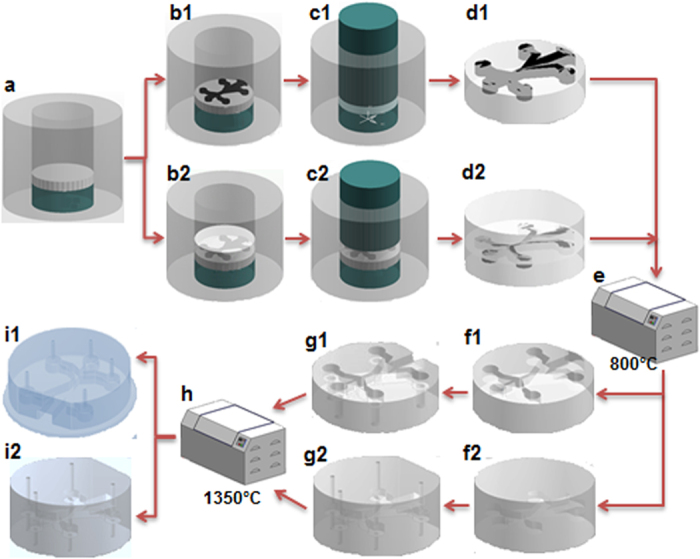
Sequence of the proposed powder processing: (**a**) powder placement; (**b**) placement of a fugitive phase (for b1 and b2) and the remaining powder (for b2); (**c–d**) powder compaction; (**e**–**f**) partially sintering and removal of fugitive phase; (**g**) drilling and other machining, (**h**–**i**) full sintering.

The overall fabrication protocols for these two configurations are depicted in Fig. 8. The open-channel configuration is denoted as ‘1’ (e.g., b1, c1, …) while the fully-enclosed configuration as ‘2’ (e.g. b2, c2, …). The fabrication of the first configuration (open-channel configuration) is only slightly different from the 2^nd^ one (fully-enclosed configuration). Thus, the fabrication protocol for the fully-enclosed micro-burner configuration is described in more details.

Alpha-phase alumina (AKP-50, Sumitomo in Japan) powder with the purity higher than 99.99% and the particle size between 0.1 and 0.3 micrometer was purchased for fabricating the proposed micro-burners. The common first step, as denoted in Fig. 8a, is to cut the 0.9 mm thick graphite sheet (EDM-3, Saturn Industries) in a CNC machine into the integrated shape of the combustion chamber and the internal channels. This graphite piece served as a fugitive phase that would later burn out during partial sintering and leave the cavities for the combustion chamber and the internal channels. Once the graphite fugitive phase was machined, a half of alumina powder (about 2 grams) was poured into the die. Prior to depositing the alumina powder, the interior of the die, whose inner diameter is 22.2 mm, was lubricated by zinc stearate (C_36_H_70_O_4_Zn) with 12.5–14% of ZnO (Alfa Aesar). This solid lubricant mixture facilitates the release of the powder compact. In addition, it also helps to reduce the frictional force that may be exerted to the powder compact with the graphite fugitive phase. After the first half of the alumina powder was deposited, the die stage was shaken with mild vibration and the punch gently pressed the powder with its own weight (~200 grams) to flatten the powder surface. Subsequently, the machined graphite was placed onto the powder surface (see Fig. 8b). The placement and orientation of the graphite piece with respect to the die reference point is crucial because it is difficult to identify the location of the cavity once the fully-enclosed cavity is formed. The other half of alumina powder was poured into the die, followed by the full compaction of the powder and the embedded graphite using MTS Insight 300 (MTS Systems Corp.) with the compaction pressure of 50MPa at a speed of 1 mm·min^−1^ (see Fig. 8c,d). The powder compact was then partially sintered in the furnace (Carbolite-HTF1700, UK) at 800 °C for 2 hours to burn out the graphite fugitive phase (Fig. 8e). The graphite reacts with oxygen in the furnace and becomes CO_2_ gas which escapes through the partially-sintered alumina compact (Fig. 8f). This partially-sintered sample with the formed cavity was drilled to make the connecting channels for fuel and oxidant sources for the micro-burner applications (Fig. 8g). The portion corresponding to the burner exhaust was milled to reveal the embedded channel. The detailed account of the machinability of the partially-sintered alumina compact will be given in a later discussion. Finally, the partially-sintered sample was fully sintered at 1350 °C to provide the mechanical integrity of the micro-burner (Fig. 8h,i).

### Material Characterization

In order to determine an appropriate partial-sintering temperature and graphite burn-out behavior, a thermogravimetric analysis (TGA Q500, TA Instruments, USA) was conducted at a constant ramping rate (15 °C·min^−1^), which is the same as the temperature ramping rate used in the proposed process. TGA measures a change in weight of the sample as a function of temperature, revealing the kinetics of graphite vaporization. During the TGA experiments, air was constantly flowing at 60 mL·min^−1^ to ensure complete oxidation of graphite. The densification process of alumina powder compact during sintering was investigated using a thermomechanical analyzer (TMA, Setaram 95, France). TMA results present the correlation between sintering temperature and sample densification kinetics in real time. A flat compacted sample was placed in between an alumina plate as a base and an alumina probe. The probe was then adjusted to zero. A change in dimension of the sample was measured by recording the movement of the alumina probe. Scanning electron microscopy (SEM) was used to evaluate the microstructures of the partially-sintered alumina samples that were sintered at different temperatures. The presence of the cavity and channels embedded in the fully-enclosed alumina sample was visualized by a computerized tomography (CT) scan (GE eXplore Locus RS micro CT) that has the highest resolution up to 27 μm.

### Micro-burner Testing Setup

Both configurations of the micro-burners (as schematically shown in Fig. 1) were prepared for the flame testing. A micro-burner with a transparent window was used to characterize the flame shape and optimize the flow rates of fuel and oxidant streams. A quartz disc (25-mm in diameter, Quartz Scientific Inc.) was bonded to the open channel device of the sintered alumina using a medium-temperature adhesive (1531 DURASEL^TM^, maximum service temperature of 343 °C). Through the quartz window attached on the channel side, an oxy-hydrogen flame can be visually observed to determine the presence and location of the flame within the chamber. The holes on the surface of the alumina micro-burner was made to insert the stainless steel (SS) tubing (0.46 mm I.D., 0.90 mm O.D.) for the fluidic connections and fixed with RESBOND^TM^ 907GF, which can endure up to 1260 °C. Once the adhesives to bond the quartz window and the SS tubing were cured at room temperature for 24 hours, the micro-burner was completed for testing. Hydrogen and oxygen were created using a commercial electrolyzer (E-65 from h-tec) coupled to the custom-made flow manifold to control the flow rates of each stream. Because the electrolyzer produces hydrogen and oxygen at a fixed stoichiometric ratio (H_2_:O_2_ = 2:1), a miniature pump was added to deliver air to the oxygen line to independently control the fuel-to-oxidant ratio. A flame image was taken using a Nikon camera (D50, shutter speed: 30 second, ISO 2.8) in a dark room. Under the normal circumstances, an oxy-hydrogen flame is not visible to bare eyes or regular cameras, and therefore the special UV intensifier would be required to image the flame. We introduced a trace amount of organic contaminants (diffusion oil) to visualize the flame.

## Additional Information

**How to cite this article**: Do, T. *et al*. Fully-Enclosed Ceramic Micro-burners Using Fugitive Phase and Powder-based Processing. *Sci. Rep.* **6**, 31336; doi: 10.1038/srep31336 (2016).

## Supplementary Material

Supplementary Information
