# Supplementary material for: Fully-Enclosed Ceramic Micro-burners Using Fugitive Phase and Powder-based Processing
Source: Sci Rep. 2016 Aug 22;6:31336. doi: 10.1038/srep31336 (PMC4992868; doi:10.1038/srep31336)
Supplement: Supplementary Information [file srep31336-s1.pdf]

Supplementary Information

Fully-Enclosed Ceramic Micro-burners Using Fugitive Phase and Powder-based Processing

Truong Do, Changseop Shin, Patrick Kwon†, Junghoon Yeom*

Surface Finish:

Ceramic processing techniques that can create embedded sub-millimeter channels or cavities (e.g. LTCC or 3D printing) in general have difficulties in controlling the surface finish of the internal features. In our proposed process, the surface finish of the inner walls is directly related to that of the graphitic fugitive phase, which is controllable through machining and other traditional processes. Figure S1 shows how two different surface finishes of the graphite (Figure S1a and S1c) can be translated into the surface finishes of the alumina walls (Figure S1b and S1d).

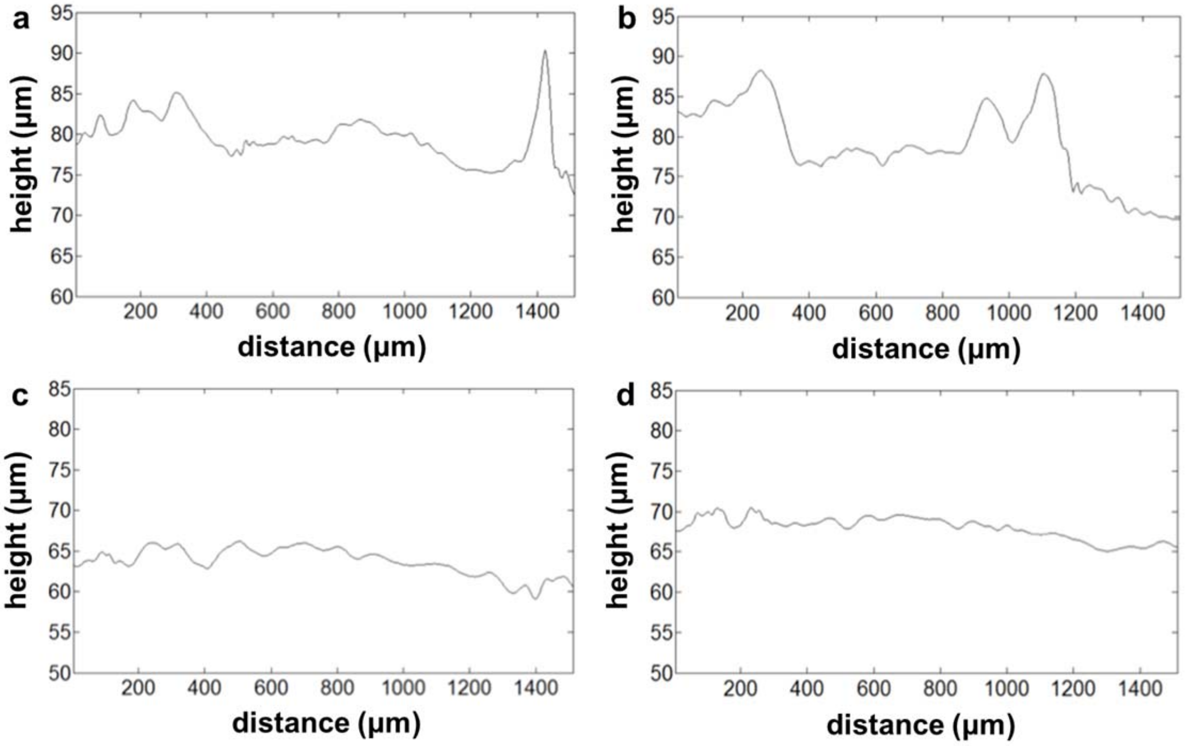

Figure S1. Profilometric scans of the graphite surfaces with two different surface finishes (a) and (c) and their corresponding alumina surface finishes (b) and (d), respectively.

Crack Monitoring:

Improper die pressing would be one of the main reasons for having cracks in the sample. In fact, the initial crack formation probably occurs at the edge or corner of the graphite during compaction regardless of the subsequent processes. For some cases, these cracks propagate to the surface of the green ceramic during compaction and can be easily spotted by bare-eye or microscope inspection (see Figure S2a). This frequently happens when the shape of the fugitive phase is distorted from what we considered to be axisymmetric. Therefore, we had to change the shape of our micro-burner design a few times to minimize macroscopic crack formation during compaction. As shown in Figure S2, we performed visual inspection at each process stage and removed the sample with the visible cracks. When the powder compact having the cracks prevalent on the exterior surface was further sintered using the optimized process (sintering temperature profile given in Figure 3b), no apparent change was seen (see Figure R2b1). Of course, there is still a possibility that cracks formed inside are not observable with visual inspection. These preformed internal cracks can propagate to the exterior surface during the sintering process. A macroscopic crack seen in Figure 3a was therefore not directly caused by the poor powder compaction, but rather by the stress(es) developed during sintering. A slower ramp rate and the addition of the soaking step to sintering alleviate the internal stress development responsible for crack propagation, leading to the crack-free sintered structure (see Figure 3b). As discussed in the paper, the crack propagation was mainly caused by the net increase in pressure within the cavity. To further explain the mechanism of how the gaseous byproducts (mainly CO_2) can be accumulated within the internal cavity during partial sintering, we provide more supporting arguments to the proposed working principle. We attribute the net increase in pressure within the cavity to the different gas permeabilities of O_2 and CO_2 in porous metal oxide structures. Generally speaking, O_2 is lighter than CO_2 and therefore exhibits a higher diffusion coefficient (D) than that of CO_2 under the same condition (for example, $D_{CO2,air}$ = 0.17 $cm^2 \cdot s^{-1}$ and $D_{O2,air}$ = 0.215 $cm^2 \cdot s^{-1}$ at 1 atm and 300 K) [S1]. More importantly, at elevated temperatures, it has been known that metal oxides are exceptionally more permeable to O_2 than other heavier gases [S2]. Therefore, for a given partial sintering condition, a higher permeability (or diffusion coefficient) of O_2 in the alumina structure provides sufficient O_2 for oxidizing a graphite fugitive phase while the byproduct CO_2 cannot easily escape from where it is produced. This issue of excessive byproduct build-up may become more severe with the decreasing pore size as the alumina structure gets consolidated further.

[S1] Ho C K and Webb S W. Gas Transport in Porous Media. Netherlands: Springer. 2006

[S2] M.V. Mundschau, X. Xie, C.R. Evenson IV, A.F. Sammells, Dense inorganic membranes for production of hydrogen from methane and coal with carbon dioxide sequestration, Catalyst Today 118 (2006) 12–23.

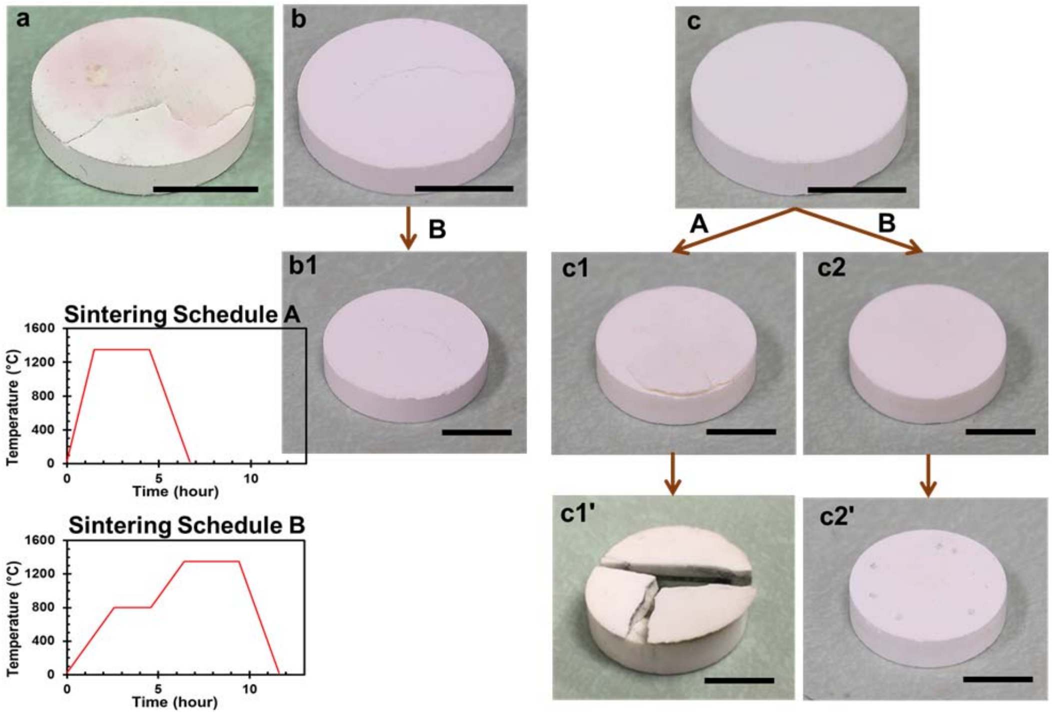

Figure S2. Photographs of the fully-enclosed alumina micro-burners at different process stages and with different sintering recipes. (a-c) green ceramics, i.e., as-pressed alumina structures; (a) the sample with the macroscopic cracks are not processed further, (b) the sample with a small, hair-like crack has been sintered using Recipe B (a slow ramp rate plus a soaking step) but the same crack has remained (b1), (c) the sample with no observable crack has been sintered using Recipe A (a fast ramp rate without a soaking step), which generates a large size crack (c1). If the sample (c) has been sintered using Recipe B, a crack-free ceramic structure can be obtained (c2). (c1') The sample (c1) has been cut in the middle to show the cross-section but shattered due to the brittleness of the fully-sintered sample. (c2') The sample (c2) has been drilled for fluidic inlets in its partially-sintered state. All scale bars are 1 cm.

Recording Dimensional Change:

Table S1. Change in the overall size and internal channel dimensions of the alumina micro-burner at each process stage

Alumina Sample Dimension (all units in mm)	Green state (after compaction)	800^oC (partial sintering)	1350^oC (% Shrinkage) (full sintering)
Overall Diameter	**22.22**	**22.14**	**18.11 (18.5%)**
Overall Thickness	**4.98**	**4.90**	**4.02 (19.2%)**
Channel Height	**0.9**	**0.89**	**0.74 (17.7%)**
Channel Width	**2.19**	**2.15**	**1.79 (18.2%)**

XRD Analysis:

The XRD (Bruker AXS D8) was carried out on the unfired, partially sintered at 600°C, 800°C, 1000°C and 1200°C samples (see Figure S3). Based on the similar peak locations of all samples, we can conclude that there is no phase change during partial or full sintering. This is expected because the starting alumina powder (without firing) is in the *alpha* phase which is stable throughout our processing route. The crystallite size (L) can be calculated from the XRD peaks using the Scherrer Equation or Modified Scherrer Equation [R4]:

$$L = \frac{K\lambda}{\beta \cos\theta}$$

where λ is the X-ray wavelength in nanometer (= 0.154 nm for Cu K-alpha), β is the peak width of the diffraction peak profile at half maximum height in radians, K is a shape factor for non-spherical crystallites (typically regarded as 0.9), and θ is the Bragg angle in radians. This equation is valid for the crystallite size smaller than 100 nm. Since the size distribution of alumina powder is from 100 to 300 nm, we can assume that the crystallites would be smaller than 100 nm and it is valid to use the Scherrer Equation. After plugging the relevant data into the equation, we obtain 8.59 nm for the average crystallite size. Since the peak widths of the diffraction signals are essentially unchanged, we can conclude that the crystallite size did not change over the samples we prepared.

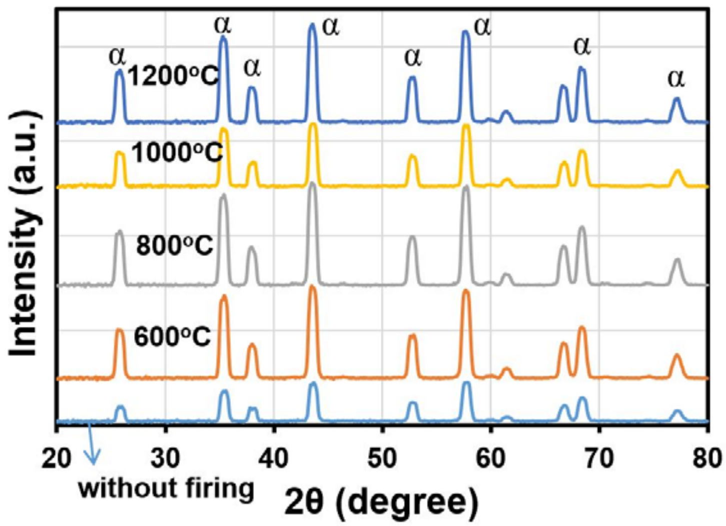

Figure S3. XRD reflection peaks from the green (unfired) ceramic and the alumina PSCs partially sintered at four different temperatures (600, 800, 1000, and 1200°C).

Flexural Strength:

We carried out a 3-point bending test to measure the flexural strength of our alumina samples. Each compacted sample was partially sintered at 600, 800, 1000 or 1200°C and then cut into a rectangular bar with the dimension of 0.5 × 1.5 × 18 mm^3. The four sides of each specimen were slightly polished by a 600-grit sandpaper to eliminate edge flaws for the testing. For each sample, the flexural strength was measured with a span length of 10 mm and a crosshead speed of 0.01 $mm \cdot sec^{-1}$ (TA Instruments RSA III, USA). The results are shown in Figure S4 and explains the trend we observed in machining PSCs. The sample partially sintered at 600°C was too fragile to machine while the sample partially sintered at 1200°C is strong but too brittle to machine. This result of the flexural strength is well correlated to the extent of neck formation observed in PSCs (see Figure 6 in the manuscript).

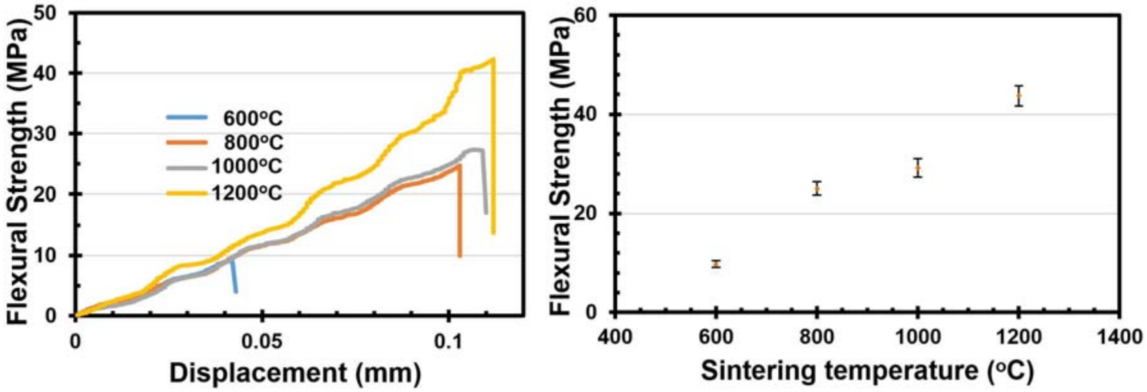

Figure S4. Plots from the 3-point bending test for PSCs with four different sintering temperatures (600, 800, 1000, and 1200°C). (Left) flexural strength vs. displacement and (Right) flexural strength vs. sintering temperature.

Micro-burner Testing:

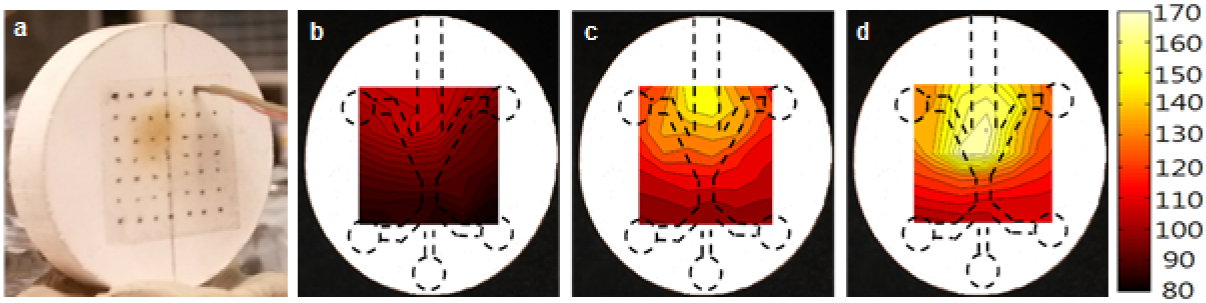

Figure S5. (a) A photograph of the 7 by 7 locations where the alumina micro-burner wall temperature was measured (burner size is 18 mm in diameter and 4.2 mm in thickness); Temperature distribution at different flow rate conditions of (b) 40 sccm of H_2, 20 sccm of O_2 + 60 sccm of air; (c) 45 sccm of H_2, 22.5 sccm of O_2 + 35 sccm of air; (d) 50 sccm of H_2, 25 sccm of O_2 + 10 sccm of air.
